# Narrative Review of Lithium Disilicate Veneers in Esthetic Dentistry: Current Perspectives and Clinical Case Report

**DOI:** 10.1155/crid/8167075

**Published:** 2026-06-25

**Authors:** Silvia Rojas-Ruedas, Franciele Floriani, Francisco X. Azpiazu-Flores, Jose Villalobos-Tinoco, Angel Gonzalez, Carlos A. Jurado, Kelvin I. Afrashtehfar

**Affiliations:** ^1^ School of Dental Medicine, Ponce Health Sciences University, Ponce, 00716, Puerto Rico, USA, psm.edu; ^2^ Department of Prosthodontics, The University of Iowa College of Dentistry and Dental Clinics, Iowa City, 52242, Iowa, USA, uiowa.edu; ^3^ Division of Restorative and Prosthetic Dentistry, College of Dentistry, The Ohio State University, Columbus, 43210, Ohio, USA, osu.edu; ^4^ Postgraduate Program in Periodontology and Implant Dentistry, School of Dentistry, National University of Rosario, Rosario, S2002KTT, Argentina, unr.edu.ar; ^5^ Independent Researcher, Culiacan, 80030, Mexico; ^6^ Department of Comprehensive Dentistry, College of Dentistry, Texas A&M, Dallas, 75246, Texas, USA, tamhsc.edu; ^7^ Department of Reconstructive Dentistry and Gerodontology, School of Dental Medicine, University of Bern, Bern, 3010, Switzerland, unibe.ch; ^8^ Oral Implantology Research Institute (OIRI), Dubai, P.O. Box: 39695, UAE

**Keywords:** adhesive, ceramic restorations, dental veneers, enamel preservation, esthetic dentistry, lithium disilicate, minimally invasive dentistry

## Abstract

**Objectives:**

To provide a focused, evidence‐based overview of lithium disilicate (LDS) veneers, with particular emphasis on indications, material properties, adhesive protocols, and clinical performance, supported by illustrative clinical cases.

**Methods:**

A narrative review of the literature was conducted to summarize current evidence on the mechanical, optical, and adhesive characteristics of LDS ceramics in veneer applications. The review emphasizes enamel preservation, adhesive reliability, and reported clinical outcomes, without undertaking a systematic comparison of study designs. In addition, two clinical case reports are presented to illustrate the clinical workflow for esthetic rehabilitation using pressed LDS veneers.

**Results:**

LDS veneers demonstrate high flexural strength (~360–400 MPa), favorable optical properties, and reliable adhesive performance when enamel is preserved and appropriate adhesive protocols are applied. Reported survival rates exceeding 95% at up to 10 years have been documented in selected patient populations. Clinical success is closely associated with appropriate case selection, conservative tooth preparation, precise adhesive procedures, and patient compliance. Common complications, including debonding, fracture, and marginal discoloration, are more frequently reported when enamel is limited or occlusal risk factors are not adequately managed.

**Conclusions:**

When applied under appropriate clinical conditions, LDS veneers represent a predictable and esthetically effective treatment option in contemporary restorative dentistry. Their long‐term success depends on meticulous technique, patient‐specific risk assessment, and adherence to evidence‐based adhesive and occlusal management principles.

## 1. Introduction

Facial esthetics, particularly dental appearance, plays a critical role in individual identity and psychosocial perception. The smile is a central component of facial attractiveness, with studies demonstrating that dental esthetics significantly influence social perception, self‐esteem, and overall quality of life [1]. A well‐aligned dentition, characterized by bright, harmonious teeth and balanced lip support, contributes to a youthful appearance and is often associated with enhanced social appeal [2].

Contemporary esthetic dentistry includes a variety of interventions aimed at improving the smile, including dental veneers, orthodontic therapy, and orthognathic surgery in cases involving skeletal discrepancies [3, 4]. These procedures not only improve facial harmony but also have a profound psychological impact on patients. Evidence indicates that individuals who are satisfied with their smile are more likely to report positive self‐perception and increased psychological well‐being [5].

Among the available esthetic restorative options, dental veneers represent a conservative and effective treatment modality for addressing color alterations, morphological discrepancies, interproximal spaces, and minor positional anomalies [6]. Veneers can be fabricated from resin‐based composite or ceramic materials, with ceramics offering superior color stability, wear resistance, and mechanical performance [7]. However, resin‐based composite veneers are more prone to discoloration and marginal degradation over time [8].

Lithium disilicate (LDS) ceramics have gained significant clinical acceptance due to their favorable combination of mechanical strength, optical properties, and long‐term stability [9]. When properly bonded to enamel, LDS ceramic veneers exhibit excellent adhesive retention and longevity, making them particularly suitable for anterior restorations where both function and esthetics are paramount. Compared to traditional feldspathic ceramics, LDS provides enhanced fracture resistance while preserving the ability to mimic natural dentition [10, 11].

The clinical performance of LDS ceramic veneers has been well documented. A retrospective clinical evaluation by Klein et al. [12] reported a 96.81% survival rate over a 10.4‐year follow‐up period, with significantly lower rates of technical and esthetic complications compared to feldspathic and leucite‐reinforced ceramics. The success of ceramic laminate veneers is highly dependent on adherence to strict clinical protocols, including case selection, conservative tooth preparation, proper surface conditioning of the ceramic and tooth substrate, and the application of appropriate adhesive systems [13]. Bond strength is significantly higher when adhesion is performed on enamel compared to dentin, emphasizing the importance of maintaining enamel preservation during preparation [14]. Given the variability in adhesive protocols based on ceramic composition and manufacturer‐specific recommendations, clinicians, particularly those early in their careers, may encounter challenges in navigating material selection and protocol adherence [15]. Therefore, understanding the principles governing adhesive cementation is essential for achieving predictable and long‐lasting clinical outcomes.

This manuscript presents a concise literature review of adhesive protocols for LDS ceramic veneers and includes two clinical case reports illustrating the workflow, decision‐making process, and clinical techniques employed in achieving a successful esthetic rehabilitation using pressed LDS veneers.

## 2. Materials and Methods

A narrative review of the literature was conducted using PubMed, Scopus, and Web of Science to identify relevant studies published up to March 2025. The search strategy combined keywords and MeSH terms related to LDS ceramics and veneer restorations, including LDS, dental veneers, esthetic dentistry, adhesive bonding, and clinical outcomes, using Boolean operators. In addition, the reference lists of selected articles and relevant reviews were manually screened to identify further pertinent publications. Given the narrative nature of the review, no formal protocol registration or quantitative synthesis was performed.

Eligible studies included peer‐reviewed clinical or translational investigations published in English that evaluated LDS ceramic veneers for anterior esthetic rehabilitation and reported outcomes such as survival rates, adhesive performance, complications, or clinically relevant material behavior. Studies were excluded if they lacked clinical relevance, provided insufficient methodological detail, or consisted solely of isolated case reports with fewer than five patients, review articles, conference abstracts, or in vitro‐only studies without clear clinical translation. Titles and abstracts were independently screened by two reviewers (Silvia Rojas‐Ruedas and Franciele Floriani), followed by full‐text assessment of potentially eligible articles. Any disagreements were resolved by consensus through discussion with a third reviewer (Kelvin I. Afrashtehfar). Extracted data included study design, sample size, preparation approach, adhesives protocol, follow‐up duration, and reported clinical outcomes or complications. Laboratory findings were incorporated where relevant to aid clinical interpretation. Potential sources of bias were addressed narratively based on study design, sample characteristics, and outcome reporting.

Two clinical cases are presented as illustrative examples to demonstrate the clinical workflow, material handling, and adhesive protocols associated with LDS veneer restorations. These cases are intended to complement the narrative review and do not represent a controlled clinical investigation.

### 2.1. First Clinical Case

**Figure 1:**
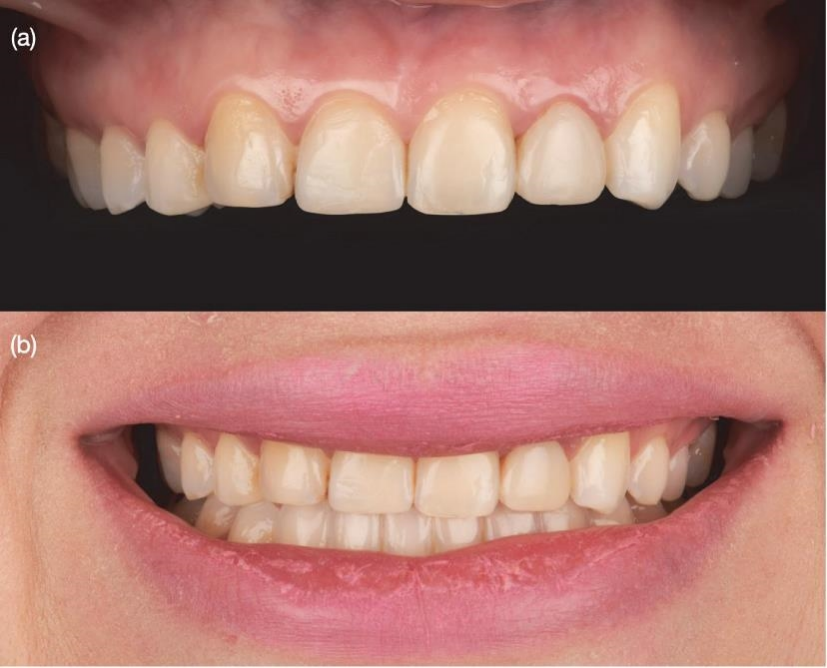
Initial situation. (a) Anterior smile view demonstrating the patient’s esthetic concerns involving the maxillary anterior teeth and (b) intraoral frontal view.

A 35‐year‐old female patient presented to the clinic with the chief complaint of wanting to improve her smile. The patient reported having had the extraction of the maxillary right canine, followed by orthodontic treatment to close the space. Upon clinical evaluation, the patient was diagnosed with mild incisal wear from the maxillary right to left canine, stained resin composite on the facial surface from the right lateral incisor to the left lateral incisor, nonideal gingival zenith positioning of the anterior teeth, and a wide smile displaying from the right second premolar to the left second premolar (Figure 1).

Several treatment options were discussed, including crown lengthening to improve gingival architecture, tooth whitening, and either direct resin composite or ceramic veneers. The patient declined surgical intervention and tooth whitening due to prior sensitivity. Given the extent of discoloration, incisal wear, and the patient’s wide smile, LDS ceramic veneers from the maxillary right second premolar to the left second premolar were proposed and accepted.

A diagnostic wax‐up (Wax GEO Classic Renfert, Hilzingen, Germany) and intraoral mock‐up were fabricated and approved by the patient. At the following appointment, partial isolation was provided with a rubber dam (Dental Dam Nic Tone, Bucharest, Romania) for the maxillary right first molar to the left first molar, secured with clamps (Clamp #00, Hu‐Friedy, Chicago, IL, USA). Minimally invasive tooth preparations were performed under partial isolation using a standardized veneer preparation system. Final tooth preparations were polished with polishing discs (Sof‐Lex XT Disc, 3M, St. Paul, MN, USA) following the recommended sequence of coarse, medium, and fine grits. After that, the ceramic veneers were digitally designed and fabricated from pressed LDS ceramic.

A double cord (Ultrapak, Ultradent, South Jordan, UT, USA) was used, and a final digital impression (Aoralscan 3, Shining 3D, Hangzhou, China) was taken for the maxilla, mandible, and both arches in occlusion. The final LDS veneers were digitally designed (Dental‐CAD 3.1, Exocad, Darmstadt, Germany) and milled from LDS shade A1 (Amber Mill, Hass Bio, Gangneung, South Korea).

**Figure 2:**
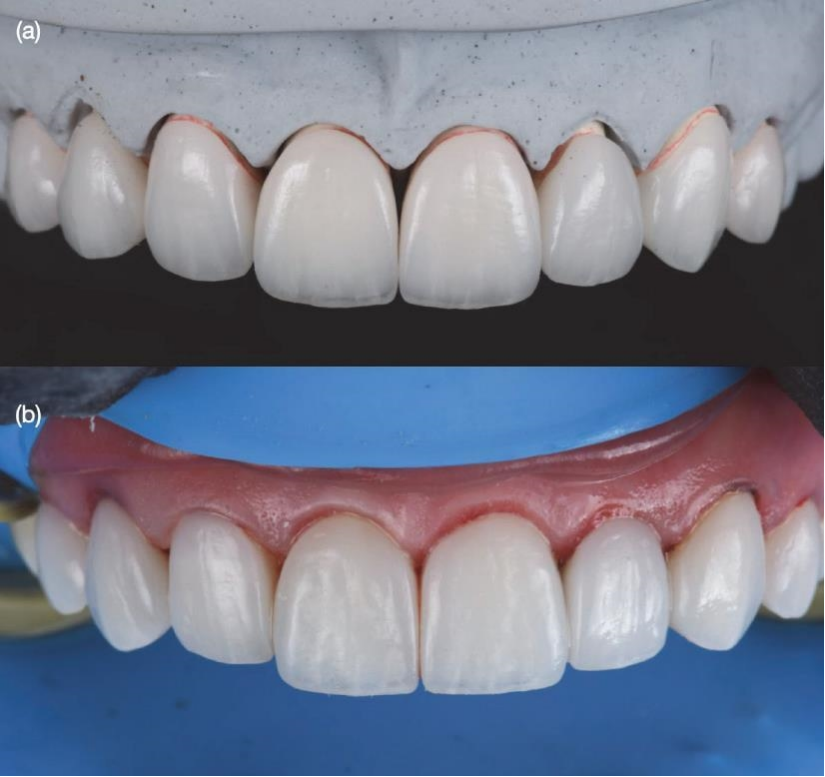
Lithium disilicate veneers on a cast model. (a) Fabricated veneers in a printed model and (b) cemented veneers under split dental dam.

The restorations were first tried in to evaluate the margins, contour, and shade, and the patient approved their cementation. Next, partial isolation was achieved using a rubber dam (Dental Dam Nic Tone, Bucharest, Romania). The ceramic veneers were then treated with 5% hydrofluoric acid (IPS Ceramic Etching Gel, Ivoclar, Schaan, Liechtenstein) for 20 s, followed by cleaning in an ultrasonic bath with 96% isopropyl alcohol for 5 min. The teeth were first sandblasted with 20 µm aluminum oxide particles and water, then etched with 37% phosphoric acid (Total Etch, Ivoclar, Schaan, Liechtenstein) for 20 s. A primer and adhesive (Optibond LF, Kerr, Brea, CA, USA) were then applied. Finally, a try‐in paste was used to evaluate the final shade. After that, the dental veneers were cemented using a light‐curing resin cement (Choice 2, Bisco Inc., Schaumburg, IL), beginning with the two central incisors, followed by the laterals, canines, and first and second premolars (Figure 2).

**Figure 3:**
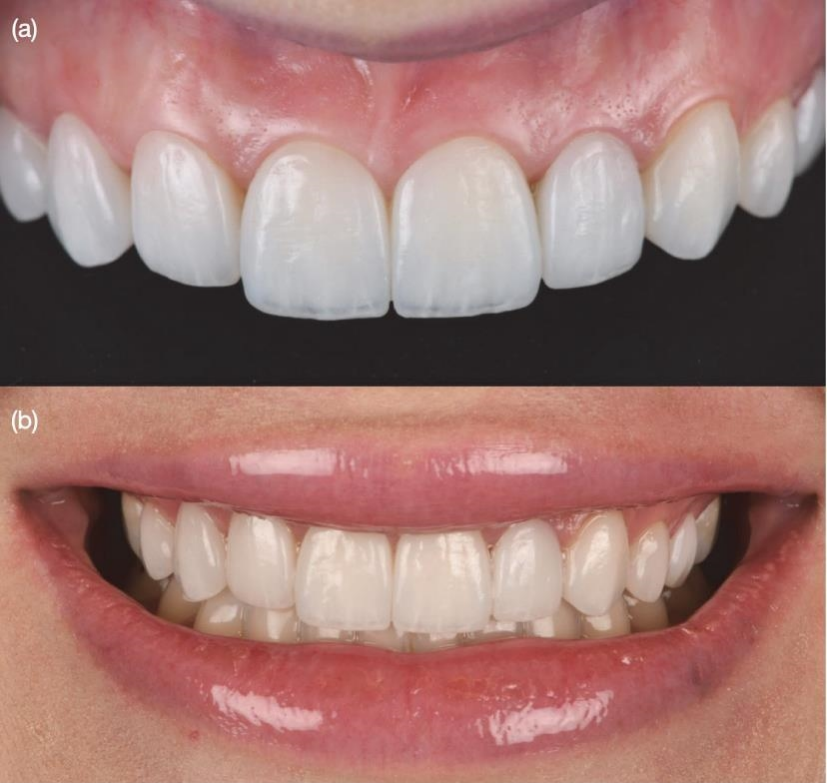
Final restorations. (a) Anterior smile view and (b) intraoral frontal view showing the maxillary final restoration.

The patient was advised to maintain good oral hygiene by brushing her teeth three times a day and was instructed to attend follow‐up visits every 6 months to evaluate the ceramic restorations and receive dental prophylaxis. The patient was satisfied with the shade and shape of the restorations (Figure 3).

### 2.2. Second Clinical Case

**Figure 4:**
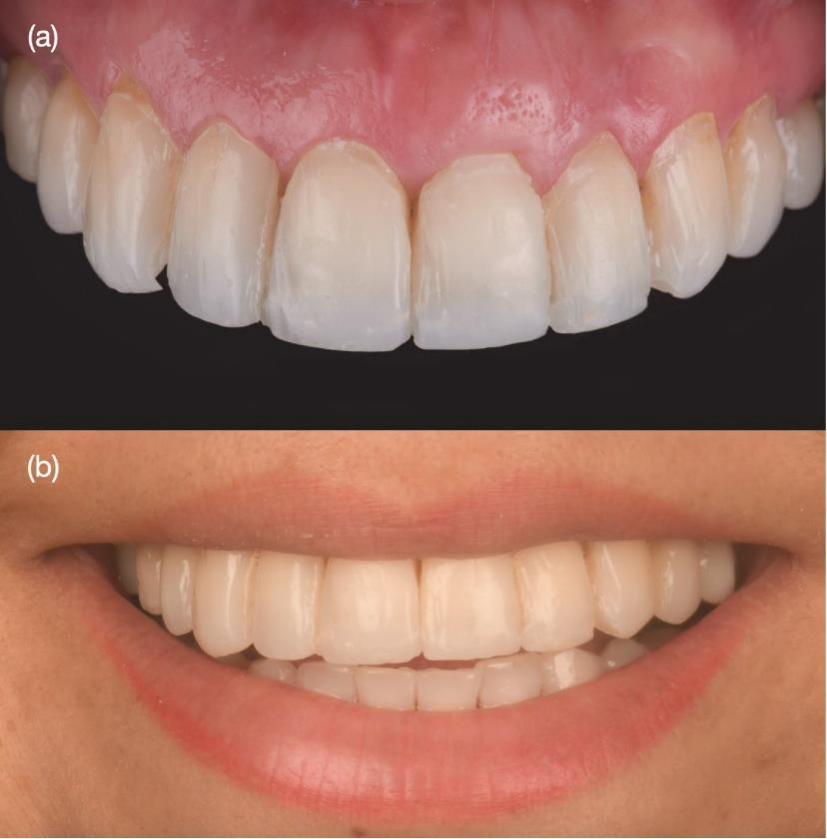
Initial situation. (a) Extraoral facial view with the patient smiling, illustrating anterior esthetics. (b) Intraoral frontal view showing the maxillary initial conditions.

A 33‐year‐old female patient presented with dissatisfaction regarding existing resin composite veneers placed 4 years earlier from the maxillary right first premolar to the left first premolar (Figure 4). Clinical examination revealed discoloration and marginal deterioration of the restorations, nonideal gingival embrasures, and incisal wear from the right to the left canines.

**Figure 5:**
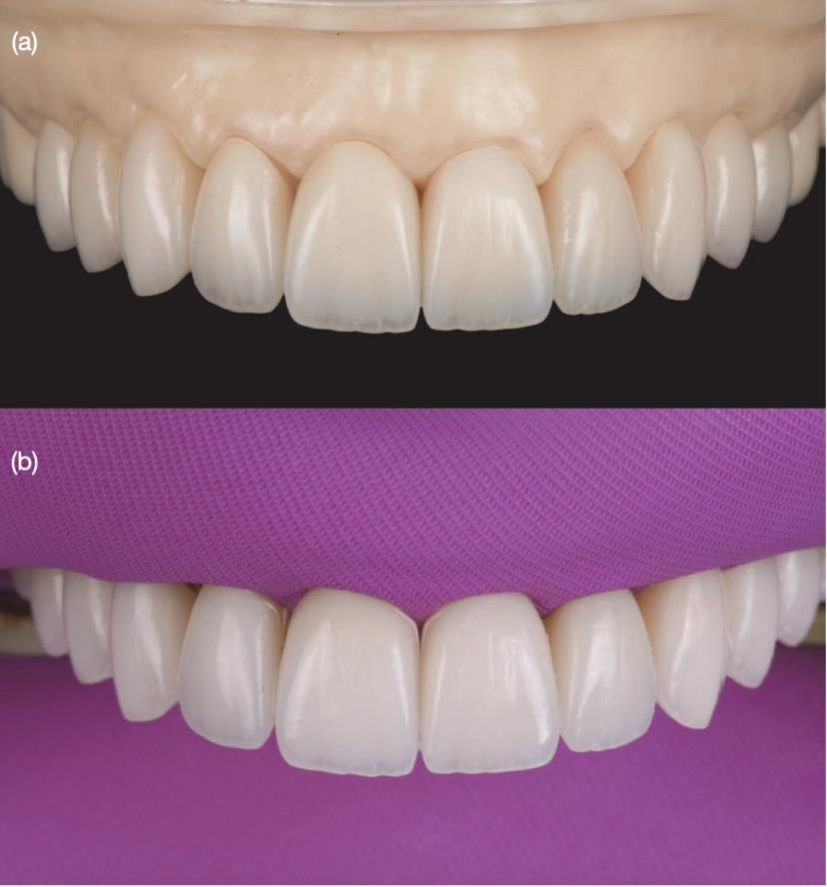
Lithium disilicate veneers. (a) Fabricated veneers in a printed model and (b) cemented veneers under dental dam.

A comprehensive treatment plan including surgical crown lengthening followed by ceramic veneers was proposed; however, the patient declined surgical treatment. Given her low smile line, restorative treatment without surgical intervention was considered acceptable. A diagnostic wax‐up and intraoral mock‐up were performed and approved. Minimally invasive tooth preparations were completed through the mock‐up, followed by finishing, polishing, gingival retraction, and digital impressions. LDS veneers were digitally designed and fabricated. The intaglio surfaces of the veneers were etched, cleaned, and silanized prior to bonding. Tooth surfaces were etched and adhesively treated, and the veneers were cemented under rubber dam isolation using a light‐curing resin cement (Figure 5). An occlusal full mouthguard was provided due to evidence of parafunctional activity.

**Figure 6:**
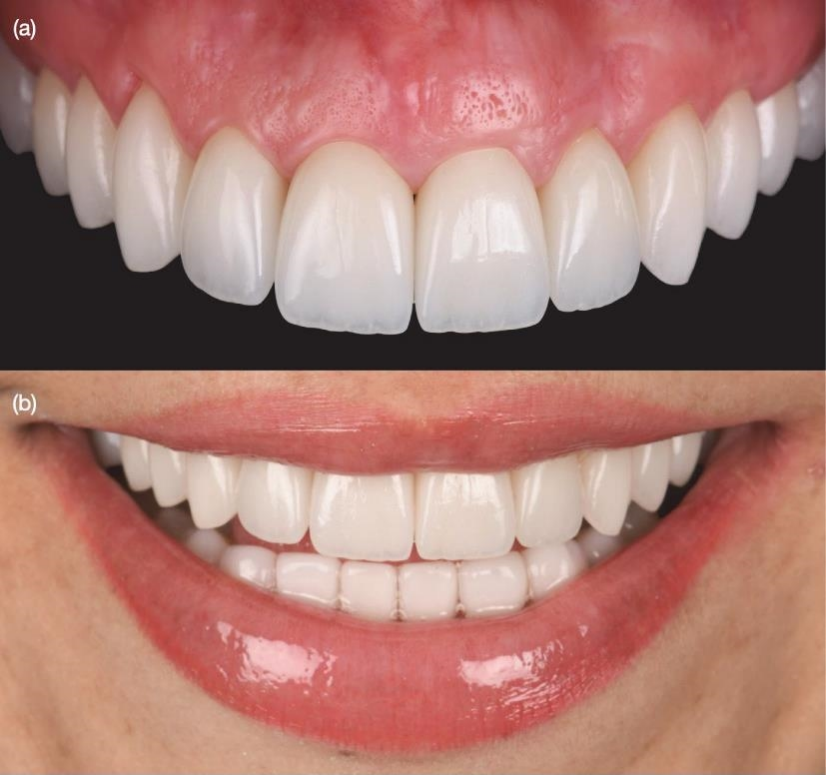
Final restorations. (a) Face smiling with final restorations and (b) intraoral frontal view with final restorations.

The patient was pleased with the final outcome of the restorations, including the shade and the shape. An occlusal full mouthguard was provided to protect the restorations at night. The patient was provided with oral hygiene instructions (Figure 6).

## 3. Results

According to this review, the success and longevity of ceramic laminate veneers rely in great part on the implementation of a precise cementation technique, starting from field isolation, adequate material selection for adhesion, proper manipulation of the materials, the seating of the veneers, polymerization, and elimination of the excess cement [16]. Several clinical steps performed before cementation, including treatment planning, preparation, impression, and adequate choice of the restorative material, could affect the quality of cementation [17]. Scientific evidence suggests careful implementation of this process to achieve predictable outcomes with ceramic laminate veneers [18]. The short‐ and long‐term clinical success of the adhesion process of ceramic veneers is tributary to a deep understanding of the materials used and the implementation of clinical protocols [19]. It is also contingent upon all the previous steps from case selection, treatment planning, and execution until and after the cementation [20].

Laboratory studies focusing on parameters predictive of the clinical efficacy of ceramic veneers, such as the tooth preparation for ceramic veneers, the selection and type of the adhesive system, the quality of marginal adaptation, the resistance against microleakage, the periodontal response, and the esthetic characteristics of the restorations, have been reviewed [21]. The clinical relevance of these parameters was then determined by reviewing the results of short‐ and medium‐ to long‐term in vivo studies involving ceramic veneers performed during the last 10 years [22]. The adhesive ceramic veneer complex has been proven to be a very strong complex in vitro and in vivo [23]. An optimal adhesion restoration was achieved especially if the preparation was located completely in enamel and if correct adhesive treatment procedures were carried out, and if a suitable resin cement was selected [24].

The maintenance of esthetics of ceramic veneers in the medium to long term was excellent, patient satisfaction was high, and ceramic veneers had no adverse effects on gingival health in patients with optimal oral hygiene [25]. Major shortcomings of the ceramic veneer system were described as a relatively large marginal discrepancy and an insufficient wear resistance of the luting composite [26]. Although these shortcomings had no direct impact on the clinical success of ceramic veneers in the medium term, their influence on the overall clinical performance in the long term is still unknown and therefore needs further study [27].

LDS veneers require a specific adhesive protocol to ensure optimal adhesion and long‐term success. The internal surface of the veneer is typically etched with 5% hydrofluoric acid for 20 s, then thoroughly rinsed and dried [28]. A silane coupling agent is applied to the etched ceramic to promote chemical adhesion between the ceramic and resin cement [29]. On the tooth side, the enamel is etched with 35%–37% phosphoric acid for 15–30 s, while dentin, if exposed, is etched selectively or treated with a self‐etch adhesive [30]. Proper isolation is critical during the adhesive process to avoid contamination. Resin cement should be chosen based on the esthetic demands and translucency of the veneer, with light‐cure options preferred for thin or highly translucent restorations due to their superior color stability [31]. LDS ceramics are well‐regarded for their superior bond strength, which contributes significantly to the durability and longevity of bonded restorations.

Their high flexural strength, typically ranging between 360 and 400 MPa, combined with excellent adhesive potential to resin cements via adhesive techniques, makes them ideal for minimally invasive restorations such as veneers, inlays, and onlays [32]. When bonded using proper adhesive protocols, LDS demonstrates exceptional fracture resistance and a strong interface between the ceramic and tooth structure, significantly enhancing clinical performance over time [33].

The etchable glass‐ceramic nature of LDS allows for efficient micromechanical retention through hydrofluoric acid etching and chemical adhesion via silanization [34]. This dual adhesive mechanism ensures a durable and reliable bond that resists degradation in the oral environment [33]. Studies have shown that restorations made with LDS exhibit higher survival rates and fewer debonding events compared to other ceramic systems [35].

LDS ceramics are renowned for their exceptional esthetics, making them one of the most preferred materials for anterior restorations [36]. Their unique microstructure, composed of fine‐grain glass‐ceramic crystals, allows light transmission and scattering properties that closely mimic natural tooth enamel and dentin [37]. This enables the creation of restorations that display lifelike translucency, brightness, and depth. The material is available in various translucency levels (HT, LT, MT, and MO), giving clinicians the flexibility to match the optical characteristics of adjacent teeth precisely while masking underlying discolorations when needed [38]. In addition to excellent translucency and fluorescence, LDS exhibits a high degree of shade stability and polishability [39]. These features contribute to a long‐lasting esthetic outcome and allow seamless integration into the patient’s smile [40].

LDS veneers have demonstrated excellent long‐term clinical performance, with multiple studies confirming their durability and stability over time [41]. Clinical survival rates for LDS veneers have consistently exceeded 90% after 10 years of function, with minimal rates of failure such as debonding, chipping, or marginal discoloration [42]. Their success is attributed to both the material’s inherent mechanical strength and its ability to be reliably bonded to enamel, creating a stable and durable restoration [43].

A 10‐year clinical study by Fradeani et al. [44] reported a cumulative survival rate of 96.3% for LDS veneers, highlighting their resilience in anterior esthetic cases. These results support LDS as a top choice for esthetic and conservative restorations, with predictable long‐term outcomes when bonded using adhesive techniques.

Both patients underwent a minimally invasive esthetic rehabilitation using a combination of full‐coverage crowns and LDS laminate veneers extending from the right second premolar to the left second premolar (Table 1). Preoperative analysis confirmed the presence of stained composite restorations, incisal wear, and asymmetry in the gingival zenith. A diagnostic wax‐up and intraoral mock‐up confirmed the feasibility of achieving the patient’s esthetic goals with conservative preparations.

**Table 1:** Clinical summary of the lithium disilicate veneer cases.

Parameter	Case 1	Case 2
Age/gender	35/female	33/female
Chief complaint	Desire to improve esthetics following orthodontic space closure	Dissatisfaction with stained and aged composite veneers
Treated teeth	Maxillary right 2nd premolar to left 2nd premolar	Maxillary right 2nd premolar to left 2nd premolar
Clinical findings	Mild incisal wear, stained composites, and low smile line	Worn, discolored composites, and gingival asymmetry
Diagnostic tools	Digital wax‐up and intraoral mock‐up	Digital wax‐up and intraoral mock‐up
Tooth preparation	Minimally invasive and enamel‐preserving	Performed over mock‐up and polished with finishing discs
Material used	Lithium disilicate (Amber Mill, shade A1)	Lithium disilicate (Amber Mill, shade A1)
Cementation protocol	HF etching, ultrasonic cleaning, silanization, enamel/dentin etch, adhesive (Optibond LF), and light‐cure resin cement (Choice 2)	HF etching, ultrasonic cleaning, silanization, enamel/dentin etch, adhesive (Optibond LF), and light‐cure resin cement (Choice 2)
Isolation technique	Rubber dam isolation (right to left first molar)	Rubber dam isolation (treated segment)
Postoperative instructions	Oral hygiene guidance and biannual follow‐up	Oral hygiene guidance, occlusal guard, and biannual follow‐up
Outcome at 1‐year follow‐up	Stable restorations, excellent esthetic result, and healthy soft tissues	Stable restorations, excellent esthetic result, and healthy soft tissues

Tooth preparation preserved enamel whenever possible, particularly in the posterior and lateral anterior teeth. The four central anterior teeth, previously restored with large composite fillings, required full‐coverage LDS crowns. All remaining teeth received minimally invasive veneer preparations with a chamfer finish line and 0.5 mm depth reduction, allowing for optimal ceramic adaptation and esthetic integration.

All 10 restorations were fabricated using the heat‐pressing technique and evaluated intraorally during a dry try‐in. Marginal adaptation, contour, and shade were clinically acceptable and approved by the patient. Cementation was completed under rubber dam isolation using a light‐cure resin cement following standardized adhesive protocols: hydrofluoric acid etching, silanization of ceramic surfaces, air abrasion of tooth surfaces, phosphoric acid etching, and application of a universal adhesive.

Immediate postoperative evaluation revealed proper seating of all restorations, with accurate marginal adaptation, harmonious emergence profiles, and successful color matching with adjacent teeth. There were no signs of cement excess, tissue irritation, or occlusal interferences. The patient expressed high satisfaction with the esthetic result.

At the 1‐year follow‐up, the restorations remained intact with no signs of debonding, chipping, marginal discoloration, or surface degradation. Gingival tissues appeared healthy and stable around all treated teeth. The patient reported no functional discomfort and demonstrated consistent use of the prescribed occlusal guard. Clinical and photographic documentation confirmed the stability of the esthetic outcome.

## 4. Discussion

The application of LDS veneers in esthetic dentistry has significantly evolved, underpinned by advances in material science and adhesive technology. The present clinical case demonstrates how a multidisciplinary approach, incorporating patient preferences, careful diagnostic planning, and evidence‐based material selection, can lead to highly satisfactory esthetic and functional results. The decision to utilize pressed LDS veneers was based not only on the patient’s desire for a conservative treatment but also on the material’s favorable performance characteristics in anterior restorations.

LDS ceramics are distinguished by their high flexural strength, translucency, and resistance to surface wear, properties that make them particularly suitable for minimally invasive veneer applications [45]. Their microstructural composition allows for the combination of esthetic finesse and structural integrity, providing a reliable solution for cases involving morphological discrepancies, discoloration, and moderate incisal wear [46]. In the present case, the use of this material was particularly advantageous given the patient’s wide smile and need for uniform shade and surface texture across multiple anterior teeth.

A central determinant of the success of dental veneers is the preservation of enamel during tooth preparation [47]. Enamel offers superior substrate characteristics for adhesives, contributing to increased retention, marginal integrity, and resistance to failure [48]. In this case, careful planning allowed for conservative reduction, maintaining sufficient enamel on most of the abutments, except for the four anterior teeth requiring full‐coverage crowns due to extensive pre‐existing restorations. This mixed restorative approach enabled a balance between esthetic goals and structural demands.

One noteworthy aspect of this case was the patient’s rejection of more invasive adjunctive procedures, such as crown lengthening, and her prior dissatisfaction with bleaching protocols. This underscores the necessity of individualized treatment planning and the value of intraoral mock‐ups and wax‐ups in establishing mutual understanding between clinician and patient. These diagnostic tools facilitated informed consent and reinforced patient engagement throughout the treatment. From a procedural standpoint, meticulous attention to adhesive protocols was critical to ensure long‐term clinical success. Surface conditioning of the LDS restorations using hydrofluoric acid and silane, followed by appropriate tooth surface treatment and adhesive selection, contributed to strong micromechanical and chemical retention [49]. The choice of a light‐cured resin cement allowed for extended working time, facilitating precise seating and cleanup, particularly in the esthetically sensitive anterior region [49].

The importance of protective measures postcementation, particularly in patients with incisal wear and possible parafunctional habits, should not be underestimated [50]. The prescription of an occlusal guard for nocturnal use in this case was a preventive strategy aimed at minimizing the risk of veneer fracture and adhesive interface fatigue [51–53]. Continued monitoring through regular follow‐up appointments was also emphasized to assess soft tissue response, occlusal stability, and restoration integrity.

While the short‐term outcomes of this case are promising, future clinical evaluation over longer periods will be necessary to confirm the durability of the restorations and detect any late‐emerging complications. Furthermore, variations in preparation geometry, enamel–dentin ratios, and occlusal schemes represent areas where additional research is warranted to optimize clinical protocols across diverse patient populations.

## 5. Conclusions

This case series demonstrates the effective application of pressed LDS restorations in the esthetic rehabilitation of a patient presenting with anterior composite failures, incisal wear, and soft tissue asymmetry. A conservative, patient‐centered approach guided by comprehensive diagnostics and precise adhesive protocols resulted in successful esthetic and functional outcomes. These findings reinforce the reliability of LDS veneers when enamel is preserved and case selection is appropriate. Long‐term success depends on patient compliance, protective measures such as occlusal guards, and regular follow‐up. Further studies are needed to validate these outcomes across diverse clinical scenarios and refine protocols for mixed complex restorative cases.

## Author Contributions

Conceptualization: Silvia Rojas‐Ruedas and Franciele Floriani. Methodology: Kelvin I. Afrashtehfar. Software: Angel Gonzalez Jr. Validation: Carlos A. Jurado, Jose Villalobos‐Tinoco, and Francisco X. Azpiazu‐Flores. Formal analysis: Silvia Rojas‐Ruedas. Investigation: Jose Villalobos‐Tinoco. Resources: Carlos A. Jurado. Data curation: Kelvin I. Afrashtehfar. Writing – original draft preparation: Franciele Floriani. Writing – review and editing: Francisco X. Azpiazu‐Flores and Kelvin I. Afrashtehfar. Visualization: Angel Gonzalez Jr. Supervision: Silvia Rojas‐Ruedas. Project administration: Angel Gonzalez Jr. Funding acquisition: Carlos A. Jurado.

## Funding

This research received no external funding.

## Disclosure

All authors have read and agreed to the published version of the manuscript.

## Ethics Statement

This study was conducted in accordance with the Declaration of Helsinki for studies involving humans. No IRB approval was required based on university guidelines.

## Consent

Written informed consent has been obtained from the patients to publish this article.

## Conflicts of Interest

The authors declare no conflicts of interest.

## Data Availability

The data that support the findings of this study are available from the corresponding author upon reasonable request.
